# Inhibition of the Exocyst Complex with Endosidin 2 Reduces Polarized Growth in *Physcomitrium patens.*

**DOI:** 10.17912/micropub.biology.000655

**Published:** 2022-11-04

**Authors:** Eric Bormann, Rholee Xu, Clare Nargi, Min Wu, Luis Vidali

**Affiliations:** 1 Department of Biology and Biotechnology, Worcester Polytechnic Institute, Worcester, MA, USA; 2 Bioinformatics and Computational Biology Program, Worcester Polytechnic Institute, Worcester, MA, USA; 3 Department of Mathematical Sciences, Worcester Polytechnic Institute, Worcester, MA, USA

## Abstract

Endosidin 2 (ES2) is a cell-permeable drug that binds to the Exo70 subunit of the exocyst complex, disrupting the final stages of exocytosis. This allows for a dose-dependent control over the process of exocytosis and greater ease in studying exocytic-dependent processes such as polarized cell growth. ES2 was utilized in studying polarized cell growth in the moss *Physcomitrium patens* , in which plants were exposed to increasing concentrations of ES2 with an IC50 between 8.8 and 12.3 µM. At 50 µM, tip-growing cells ruptured close to their tips, an indication that ES2 inhibits the deposition of new cell wall material via exocytosis. This data serves to further support the use of ES2 as a tool to interfere with exocytosis with lethality only seen at high levels of ES2.

**Figure 1. Dose-response of moss protonemata polarized growth to Endosidin-2, and tip cell wall rupture at the high dose:**
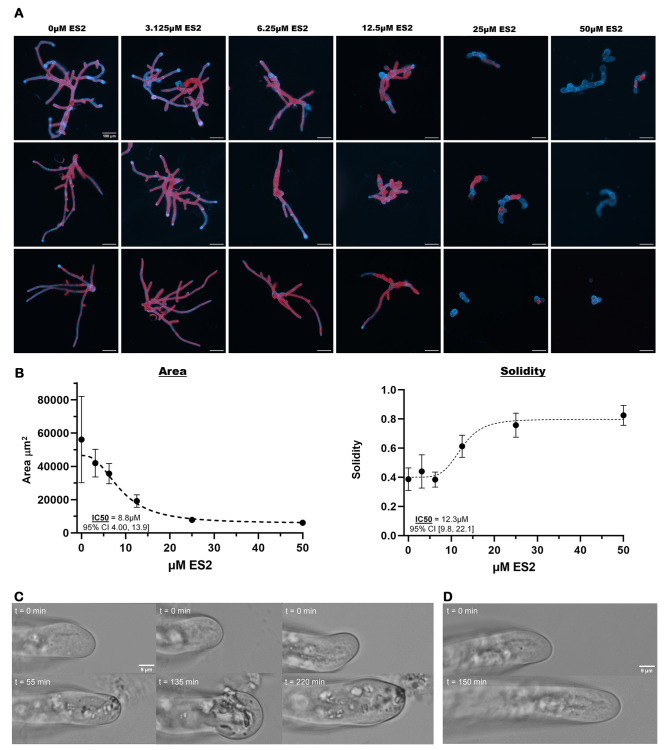
(A) Images of individual wild-type plants grown on PPNH4 media containing various concentrations of Endosidin 2 (0 µM to 50 µM). The blue shows the cell walls, and the red shows the chlorophyll of the plants. All scale bars are set to 100 µm. (B) Graphs of the average area and solidity of the plants. Each point on the graph represents the average from four growth assay experiments, with each experiment having 10-30 plants per plate that were measured, with the error bars showing the range of values in each treatment. The average area of the plants can be seen decreasing as ES2 exposure is increased, showing a decrease in overall growth. The average solidity of the plants can be seen increasing with exposure to ES2, showing that the polarized cell growth is specifically decreasing. The calculated IC50 of the dose-response and 95% confidence interval of the IC50 were also calculated using Graphpad Prism. (C) Growing *P. patens* caulonema cells rupture after treatment of Endosidin2. Three representative tip cells growing normally before treatment of 50 µM ES2 in 0.1% DMSO at time t = 0 min. (D) Control experiment of a representative tip cell growing normally before treatment of 0.1% DMSO at time t = 0 min. After 150 minutes, no tips were ruptured, and cells continued to grow normally for several hours.

## Description

Polarized cell growth is an essential eukaryotic growth mechanism allowing for unidirectional cell growth. Examples of polarized growth can be seen in neuronal axons and dendrites of animals, in fungal hyphae, and in root hairs and pollen tubes of plants (Richter et al., 2011). Polarized growth is also key to the development of moss plants (Prigge & Bezanilla, 2010). For polarized growth to function properly, cells must tightly control membrane trafficking. One of the key components that must be regulated is the exocyst complex, an octameric protein complex that tethers secretory vesicles to sites of membrane fusion, allowing for cell growth (Lepore et al., 2018; Mei & Guo, 2018). Numerous inhibitors and Endosidins that target the endomembrane system have been identified in screenings using *Arabidopsis thaliana* (Drakakaki et al., 2011). One of these Endosidins identified is Endosidin 2 (ES2), a cell-permeable exocytosis inhibitor that targets the Exo70 subunit of the exocyst complex. This allows disruption of the exocytic pathway while avoiding the lethality that mutants would introduce to organisms when interfering with the exocyst complex (Zhang et al., 2015). Further studies of the effect of ES2 have shown that it also affects PINs, disrupting trafficking of PIN2, PIN3, and PIN4; however, the function of SYT1 was observed to be unaffected in *A. thaliana* (Lešková et al., 2020). SYT1 is a member of the synaptotagmin family, and its proper function is essential for cell viability in *A. thaliana* (Schapire et al., 2008).

Here, we show that exposure to ES2 inhibits polarized growth in wildtype *Physcomitrium patens* , with higher levels of exposure having a greater impact on polarized growth. Plants not exposed to any ES2 had an average area of about 56,000 μm ^2^ and an average solidity (defined as the ratio of the plant’s area over the plant’s convex-hull area) of 0.39. As ES2 concentration exposure increased, the area of the plants decreased, and the solidity increased so that at 50 µM ES2 exposure, the average area was 6,000 μm ^2^ with an average solidity of 0.83. Using the average area values, the IC50 of Endosidin 2 for wildtype *P. patens* was found to be 8.8 µM with a 95% confidence interval between 4.0 µM and 19.9 µM. Using the average solidity values, the IC50 was found to be 12.3 µM with a 95% confidence interval between 9.2 µM and 22.1 µM. At exposure levels much greater than calculated IC50s (25 and 50 µM), little to no red fluorescence could be seen, indicating that there is no chlorophyll present in the plants and that the plants are dead or dying from the exposure to ES2. Treating elongating cells with 50 μM results in cell wall rupture, causing loss of cytoplasm and cell death. Tips ruptured at varying times after treatment, with an average rupture time of 140 minutes taken across 15 cells and 3 separate experiments. Slight swelling at the tip also occurred in most cells before bursting. Because cell wall materials and cell wall synthesizing enzymes are deposited by exocytosis, the cell wall rupture likely results from a loss of exocytosis.

Exposing *P. patens* to ES2 had a significant impact on the polarized growth of the plants, with the IC50 calculated to be between 8.8 and 12.3 µM. This calculated IC50 is similar to the IC50s of ES2 and the more potent analog, ES2-14, which were found to be 32 and 15 µM in *Arabidopsis thaliana,* respectively (Huang et al., 2019), with the lower IC50 possibly indicating that *P. patens* is more sensitive to ES2 than *A. thaliana* . The decrease in polarized growth from ES2 exposure is most likely explained by the interaction of ES2 with Exo70 preventing proper binding between the exocyst complex and PtdIns(4,5)P _2_ . The current model of plant polarized growth shows that the exocyst complex must bind to PtdIns(4,5)P _2_ to allow for vesicles to fuse to the plasma membrane, an interaction believed to be mediated by the Exo70 and Sec3 exocyst subunits due to the high affinity these subunits have for PtdIns(4,5)P _2_ (Liu et al., 2007; Orr et al., 2020; Zhang et al., 2008). Additionally, with ES2 known to disrupt PINs in *A. thaliana* , auxin trafficking in *P. patens* is likely to be disrupted, affecting the proper signaling for cell growth; however, we do not believe this to be the primary cause of decreased growth. Lethality of *P. patens* was observed at high levels of ES2 exposure, the lethality is likely not due to depletion of the moss’ SYT1 activity as ES2 does not affect SYT1, and membrane integrity is able to be maintained (Lešková et al., 2020; Schapire et al., 2008). Based on our results, we propose that lethality is due to cell wall bursting due to reduced secretion of cell components. Overall, Endosidin 2 is a viable tool to induce dose-dependent responses for studying the exocyst complex and polarized growth in plants.

## Methods

Week old *P. patens* tissue was harvested and protoplasted in a mixture consisting of 0.5% driselase in 8% mannitol. After sitting in the driselase and mannitol mixture for one hour on a shaker table while covered, sieved using a 70 µm cell strainer to remove large debris and select for single protoplasts. The remaining mixture was centrifuged for 5 minutes on a low setting (500 g) to avoid damaging the protoplasts, the supernatant was discarded, and the protoplasts were washed with 8% mannitol, a total of three times before the cell concentration was calculated manually using a hemocytometer. The protoplasts were then suspended in liquid PPNH4 + 8% mannitol with 10 mM CaCl _2_ at a concentration of 50,000 cells/ml. 150 µl aliquots containing 7,500 cells were taken and plated onto PRMB media covered by cellophane sheets. The newly seeded plates were placed into a growth chamber set to 25°C with a 16-hour light and 8-hour dark cycle for four days. After four days, each cellophane sheet was transferred to ES2-treated PPNH4 media. To prepare the treated media, 5mg of ES2 purchased from Cayman Chemical was dissolved in 241.4 µl of DMSO to create a 50 mM stock. All excess ES2 was stored at -20°C. The treated media had final ES2 concentrations of 50 µM, 25 µM, 12.5 µM, 6.25 µM, and 3.125 µM. In addition to the ES2-treated media, a control media containing 0.1% DMSO was also made. The exact volumes of ES2 and DMSO added to 16 ml aliquots of melted PPNH4 are as follows: 50 µM: 16 µl ES2, 25 µM: 8 µl ES2 and 8 µl DMSO, 12.5 µM: 4 µl ES2 and 12 µl DMSO, 6.25 µM: 2 µl ES2 and 14 µl DMSO, 3.125 µM: 1 µl ES2 and 15 µl DMSO, and 0.1% DMSO: 16 µl DMSO and 0 µl ES2.

After transfer to the drug-treated media, the plants were returned to the growth chamber for three more days before imaging. The cellophanes from the drug-treated media containing the plants were removed and placed plant-side down onto a thin layer of PPNH4 media containing 10 mM calcofluor. Using an automated epifluorescence microscope with a 10x lens and a NA of 0.3, a 12x12 grid of images was taken and stitched together into a single image using the blue fluorescence of the cell wall to align the frames (Zen software Zeiss). Stitched images were run through a custom ImageJ macro ( **Growth_assay_V7.ijm** ) to calculate the area, solidity, and chlorophyll values of all the plants that can be seen in their entirety within the stitched image. The edited output from this macro was then manually curated to remove any artifacts there were counted as plants by the ImageJ macro. The curated data was then run in a custom R-code ( **Endosidin_growth_V2.R** ), in which all plants of a minimum size (determined by selecting the size of one of the smallest living plants) and plants of a maximum size (larger than three times the standard deviation) were excluded. The output of the R-code was exported to GraphPad Prism for final graphing and analysis.

One week old *P.patens* protonemal cells grown in W-Pi media were harvested and added to a microfluidic device. The cells were then adapted in the chamber for 24 hours with a constant flow of liquid media. Cells were monitored at 20 hours to ensure consistent tip growth. After 24 hours, 1 ml of 50 µM ES2 was added to the chamber, and flow was stopped. Tip cells were imaged at 40X (Zeiss AxioObserver) every 5 minutes after ES2 addition for 4 hours. The control experiments were identical, except for the addition of 1 ml of 0.1% of DMSO instead. Microfluidic chambers were ~20 µm tall with a 60 µm tall loading area, casted with PDMS. Chambers were then plasma bonded to 35 mm glass bottom Petri dishes (MatTek).
